# Focus on the Role of Klotho Protein in Neuro-Immune Interactions in HT-22 Cells Upon LPS Stimulation

**DOI:** 10.3390/cells9051231

**Published:** 2020-05-16

**Authors:** Kinga Rusinek, Przemysław Sołek, Anna Tabęcka-Łonczyńska, Marek Koziorowski, Jennifer Mytych

**Affiliations:** Department of Animal Physiology and Reproduction, Institute of Biology and Biotechnology, Collegium Scientarium Naturalium, University of Rzeszow, Werynia 2, 36-100 Kolbuszowa, Poland; rusineczek96@gmail.com (K.R.); pp.solek@gmail.com (P.S.); annaurz@wp.pl (A.T.-Ł.); mkozioro@ur.edu.pl (M.K.)

**Keywords:** klotho, lipopolysaccharide, HT-22 hippocampal neuronal cells, neuroinflammation, ER stress

## Abstract

Neuroinflammation is defined as the activation of the brain’s innate immune system in response to an inflammatory challenge and is considered to be a prominent feature of neurodegenerative diseases. The contribution of overactivated neuroglial cells to neuroinflammation and neurodegenerative disorders is well documented, however, the role of hippocampal neurons in the neuroinflammatory process remains fragmentary. In this study, we show for the first time, that klotho acts as a signal transducer between pro-survival and pro-apoptotic crosstalk mediated by ER stress in HT-22 hippocampal neuronal cells during LPS challenge. In control HT-22 cells, LPS treatment results in activation of the IRE1α-p38 MAPK pathway leading to increased secretion of anti-inflammatory IL-10, and thus, providing adaptation mechanism. On the other hand, in klotho-deficient HT-22 cells, LPS induces oxi-nitrosative stress and genomic instability associated with telomere dysfunctions leading to p53/p21-mediated cell cycle arrest and, in consequence, to ER stress, inflammation as well as of apoptotic cell death. Therefore, these results indicate that klotho serves as a part of the cellular defense mechanism engaged in the protection of neuronal cells against LPS-mediated neuroinflammation, emerging issues linked with neurodegenerative disorders.

## 1. Introduction

Neuroinflammation is defined as a complex inflammatory response within the central nervous system (CNS) mediated by the expression of several inflammatory molecules (cytokines, chemokines, reactive oxygen species). Despite initial consideration of CNS being an immune-privileged site, current data suggest that not only neuroglia but also neuronal cells may be both immune-competent and interact with the peripheral immune system. In response to a variety of insults, neuronal cells have been shown to initiate activation of NF-κB transcription factor and stimulate intensive inflammatory response [1]. Separate studies indicate that neurons may also directly regulate the effector functions of CNS-infiltrating T cells in the absence of specialized antigen-presenting cells [2]. These interactions on the line immune-nervous system, but also local ‘immune’ functionality of CNS, are crucial for an effective response to foreign antigens and maintaining the global homeostasis of CNS [3]. Furthermore, the degree of neuroinflammation depends on the context, duration, and course of the primary stimulus, and in stark contrast to the older view, neuroinflammation is now realized to have both neuroprotective as well as neurotoxic functions [3,4,5]. Extensive neuroinflammation has been considered to be a prominent feature of neurodegenerative diseases such as Alzheimer’s disease, Parkinson’s disease, multiple sclerosis, motor neuron disease, ischemia and traumatic brain injury, depression, and autism spectrum disorder [6]. While the contribution of overactivated neuroglial cells to neuroinflammation and neurodegenerative disorders is well documented [7,8], the knowledge about the role of hippocampal neurons and factors affecting neuronal functionality during the inflammatory process is limited to a few already mentioned reports [1,2,9]. 

Klotho was originally characterized as a putative age-suppressing gene. It is expressed primarily in the kidneys and brain choroid plexus (a region in close proximity to hippocampus) [10]. Several alterations in CNS function have been described in klotho deficient mice, i.e., behavioral impairments, synaptic loss, hypomyelination, and increased phosphorylation of neurofilaments [11]. Klotho reduction in choroid plexus triggers inflammation and initiates activation of innate immune cells within an adjacent brain region following a peripheral immune challenge. Furthermore, klotho suppresses activation of the NLRP3 inflammasome in macrophages by enhancing FGF 23 signaling, and thus, acts at the interface between the brain and immune system [12]. However, the role of klotho protein in the response of hippocampal neurons to neuro-inflammatory stimulus remains unclear. 

Thus, the purpose of this study was to determine the effect of klotho-silencing on mouse hippocampal neuronal cells (HT-22 cell line) exposed to LPS. This study will bring new important insight into mechanisms of neuroinflammation, emerging issues linked with neurodegenerative disorders.

## 2. Materials and Methods

### 2.1. Materials

The reagents used, if not mentioned otherwise, were purchased from Sigma (Poznan, Poland) and were of analytical grade. Antibodies’ catalog numbers, their RRIDs, and primer sequences are presented in the supplement. 

### 2.2. Cell Culture, KLTH Silencing, and LPS Treatment

Mouse hippocampal neuronal cells (HT22; Thermo Fisher Scientific, Waltham, MA, USA) (3.0 × 10^3^ cells/cm^2^) were cultured at 37 °C in Dulbecco’s Modified Eagle’s medium (DMEM) with 4.5 g/L glucose and 1 mM sodium pyruvate (without L-glutamine) supplemented with 10% fetal bovine serum and 1% antibiotic and antimycotic mixed solution (100 U/mL penicillin, 0.1 mg/mL streptomycin, 0.25 µg/mL amphotericin B and 29.2 mg/mL L-glutamine) in a humidified atmosphere in the presence of 5% CO_2_ until they reached 80% confluence. Typically, cells were passaged every 3 days by trypsinization and maintained in DMEM. For klotho downregulation, cells were seeded at a density of 7.5 × 10^3^ cells/cm^2^ into a 12-well plate and after 24 h, transfected with 10 pmol siRNA (suspended in 3 µL DMEM without FBS) using 3 µL of Lipofectamine RNAiMAX reagent and following standard manufacturer’s instructions. siRNAs used were: Ctrl-siRNA (Silencer Negative Control No. 1 siRNA; #AM4611, Thermo Fisher Scientific) or KLTH-siRNA (UniGeneID Hs.524953; siRNA ID 15204; #AM16706, Thermo Fisher Scientific) [13,14,15]. Silencing of klotho was confirmed with the Western Blot method. For the experiments, two days after transfection, cells were trypsinized, seeded (3.0 × 10^3^ cells/cm^2^) and after 24 h, treated with 1 µg/mL LPS (prepared in PBS) and incubated for another 48 h. The LPS concentration and exposure time were selected based on the literature [16,17,18,19].

### 2.3. MTT Assay 

For the MTT assay, a standard protocol reported in the previous study was employed [20]. After 48 h incubation with or without LPS, the cell culture medium was replaced with medium supplemented with MTT (0.5 mg/mL). After 4 h incubation, the medium was removed and the formed crystals were dissolved in DMSO. Absorbance was read at 570 nm (measurement wavelength) and 630 nm (reference wavelength) with a microplate reader Victor X4 (Perkin Elmer, Waltham, MA, USA) and the results are presented as %, while MTT activity at standard growth conditions is considered as 100%.

### 2.4. ATP Level Determination 

ATP concentration was measured using ATPlite Luminescence Assay (Perkin Elmer) according to the manufacturer’s protocol. Briefly, the cell culture medium was removed, the cells were lysed with lysis solution and the substrate solution was added to generate a luminescent signal, which was further monitored with a microplate reader (Victor X4, Perkin Elmer, Waltham, MA, USA). The results are presented as relative luminescent units (RLU).

### 2.5. Morphological Analysis and Cytoskeleton Staining

Cellular morphology was controlled using Zeiss Axiovert 40CFL (Oberkochen, Germany) inverted microscope and a computer image analysis system Zeiss Axiovert 40CFL. To visualize the cytoskeleton structure, tubulin was stained using Tubulin Tracker fluorescent dye (Thermo Fisher Scientific). The cell culture medium was discarded, cells were washed twice with Hanks’ Balanced Salt Solution (HBSS) and supplemented with 50 nM working solution of Tubulin Tracker prepared in HBSS. After 30 min incubation at 37 °C in the dark, cells were rinsed twice with HBSS and digital cell images were captured with an InCell Analyzer 2000 (GE Healthcare, Chicago, IL, USA) equipped with a high-performance CCD camera.

### 2.6. Oxidative and Nitrosative Stress Parameters

Intracellular superoxide production, reduced glutathione level and nitric oxide pool were estimated using the fluorogenic probes [dihydroethidium, Thiol Tracker Violet, Thermo Fisher Scientific (Waltham, MA, USA) and 4-amino-5-methylamino-2′,7′-difluorofluorescein diacetate, Cayman Chemicals (Ann Arbor, MI, USA), respectively]. Briefly, the cell culture medium was removed, cells were washed twice with PBS and covered with PBS supplemented with a mixture of fluorogenic probes (5 µM of each). After 15 min incubation at room temperature in the dark, cells were rinsed twice with PBS and digital cell images were captured with an InCell Analyzer 2000 (GE Healthcare, Chicago, IL, USA). Quantitative analysis was conducted with the InCell Analyzer analysis module and data are presented as relative fluorescence units (RFU). 

### 2.7. Measurement of Intracellular Calcium and Zinc Levels

Fura-PE3AM and Zinquin ethyl ester (Cayman Chemical, Ann Arbor, MI, USA) were used to control the pools of intracellular calcium and zinc, respectively. Cells were trypsinized, washed twice with PBS, suspended in HBSS (Zinquin ethyl ester) or PBS *w*/Ca^2+^Mg^2+^ (Fura-PE3AM) at the density of 2.0 × 10^5^ cells/mL and 5 µM of the fluorescent probe was added. The fluorescence intensity was measured in a microplate reader (Victor X4, Perkin Elmer, Waltham, MA, USA) (λex = 485 nm, λem = 535 nm) after 15 min incubation at room temperature in the dark. The data are presented as %, while the level of calcium/zinc at standard growth conditions is considered as 100%.

### 2.8. Cell Cycle and Micronuclei Formation Analysis 

For cell cycle status and micronuclei formation evaluation, HT22 cells were rinsed with PBS, fixed in 4% paraformaldehyde and stained with 1 µg/mL Hoechst 33,342 for 20 min at room temperature in the dark. The nuclei visualization was performed using an InCell Analyzer 2000 (GE Healthcare, Chicago, IL, USA). ImageJ software equipped with DNA Cell Cycle plug-in was used to assess the cell cycle profile and the results are presented as % of cells in G0/G1, S and G2/M phases. For micronuclei analysis, a minimum of 1000 cells were examined for each sample and results are presented as % of micronuclei of total cells.

### 2.9. Enzyme-Linked Immunosorbent Assays (ELISAs)

The ELISAs were performed using commercially available kits (Thermo Fisher Scientific, Waltham, MA, USA) according to the manufacturer’s protocol. Briefly, cell culture media were collected, loaded onto ELISA plates together with a blocking solution and incubated for 3–4 h. After washing, primary antibodies HRP-labeled were added and incubated for another 1–2 h. The signal was detected using TMB substrate. The readouts were done using a microplate reader (Victor X4, Perkin Elmer, Waltham, MA, USA) and results are presented as pg/1000 cells.

### 2.10. Acridine Orange Staining

A standard protocol reported in the previous study was employed for acridine orange staining [13]. In general, cells were washed twice with PBS and covered with DMEM supplemented with 1 µg/mL acridine orange. After 15 min incubation at 37 °C in the dark, cells were rinsed twice with PBS and digital images were captured with InCell Analyzer 2000 (GE Healthcare, Chicago, IL, USA). Quantitate analysis was conducted using the InCell Analyzer analysis module and presented as relative fluorescent units (RFU).

### 2.11. BrdU Incorporation Assay and Immunostaining 

To assess proliferation rate, 10 µM BrdU was added to cell culture medium 4 h before fixation. Then, the standard protocol described elsewhere was used [21]. Briefly, cells were fixed with 4% paraformaldehyde for 15 min, permeabilized for 20 min with PBS-T (PBS supplemented with 0.25% Triton-X), denatured with 2 M HCl for 1 h at 37 °C, blocked with 1% bovine serum albumin (BSA) and incubated overnight with primary antibody prepared in 1% BSA. After that, cells were washed twice with PBS-T and incubated for 1 h with fluorochrome-conjugated secondary antibody. Nuclei were visualized with 1 µg/mL Hoechst 33,342. Digital images were taken with the InCell Analyzer 2000 (GE Healthcare, Chicago, IL, USA). For BrdU incorporation, a minimum of 1000 cells were examined for each sample and results are presented as % of BrdU positive cells of total cells. Denaturation with HCl was omitted in the protocol for H2A.X immunostaining. H2A.X formation was quantitatively analyzed using ImageJ software and presented as puncta/cell. For surface immunostaining (CD extracellular expression), all buffers used did not contain Triton-X. Quantitate analysis of CD expression was conducted using an InCell Analyzer analysis module and presented as relative fluorescent units (RFU). All antibodies’ details are presented in the supplement. 

### 2.12. RNA Purification, cDNA Synthesis, and PCRs

Total cellular RNA was extracted using TRIzol reagent (Thermo Fisher Scientific, Waltham, MA, USA) according to the manufacturer’s protocol. The concentration and purity of RNA were controlled spectrophotometrically at an optical density of 260/280, while its integrity during 1.5% agarose electrophoresis. Then, 2 µg of RNA was reverse transcribed using High-Capacity cDNA Reverse Transcription Kit (Thermo Fisher Scientific, Waltham, MA, USA) strictly following the attached instructions. For PCR reactions, the reaction mixture containing 5 µL of 2 × PCR TaqNova-RED Master Mix (DNA Gdansk, Gdansk, Poland), 4 µL of primers (2 µL of each 1 µM forward and reverse primer, Genomed, Warsaw, Poland) and 1 µL of cDNA (10 ng) was prepared. The PCR reactions were conducted for 35 denaturing cycles at 95 °C for 45 s, annealing at the temperature designated for each primer pair for 45 s, followed by extension at 72 °C for 45 s, and a final extension at 72 °C for 10 min. Primer sequences are provided in Tables S1 and S2 in Supplementary File. PCR products were resolved during 1.5% agarose electrophoresis. Densitometry analysis was conducted with GelQuantNET and results are presented as the relative density normalized to the expression level of housekeeping gene *ACTB*.

### 2.13. Western Blotting

The Western Blot protocol was described previously [22,23]. In general, cells were lysed in RIPA buffer and protein concentration was determined with a BCA Assay Kit (Thermo Fisher Scientific, Waltham, MA, USA). Then, 30 µg of proteins were separated by 10% SDS-PAGE and electroblotted to PVDF membranes. Further to this, membranes were blocked in 3% BSA (for anti-klotho antibody) and 1% BSA (for the rest of the antibodies), incubated with the primary antibodies and HRP-conjugated secondary antibody. Immunocomplexes were visualized using the ECL substrate and Fusion Fx7 system. The relative protein expression levels were normalized to the levels of ACTB (GelQuantNET software).

### 2.14. Statistical Analysis

The results represent the mean ± standard deviation (SD) from at least three biologically independent experiments (*n* = 3). The data were analyzed with 1-way ANOVA followed by Dunnett’s multiple comparison test. A p-value of < 0.05 was considered as statistically significant (***/^^^ *p* < 0.001; **/^^ *p* < 0.01; */^ *p* < 0.05, no indication/no statistical significance). (*) indicates a comparison between LPS-untreated and treated Ctrl-siRNA or KLTH-siRNA cells, (^) indicates a comparison between LPS non-treated Ctrl-siRNA and KLTH-siRNA cells, or LPS-treated Ctrl-siRNA and KLTH-siRNA cells

## 3. Results

### 3.1. Klotho-Depleted HT-22 Hippocampal Neuronal Cells are Sensitive to LPS Stimulation

**Figure 1:**
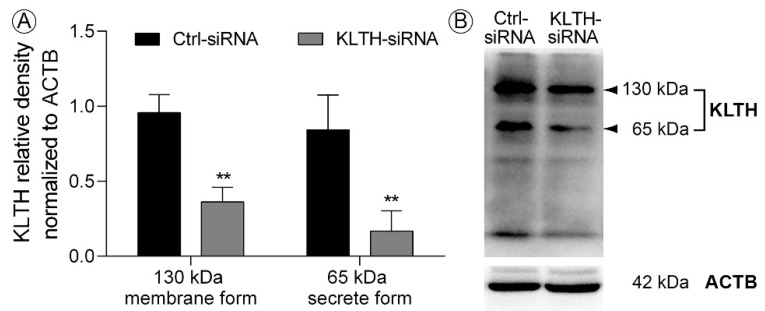
siRNA mediated depletion of klotho in HT-22 hippocampal neuronal cells (**A**) Western Blot analysis of klotho membrane and secreted forms expression after transfection; (**B**) representative Western Blot. Bars indicate SD, *n* = 3, ** *p* < 0.01 (one-way ANOVA and Dunnett’s a posteriori test).

Klotho was silenced using siRNA strategy with a similar result in HT-22 mouse hippocampal neuronal cells as presented elsewhere [14]. As described previously, transfection with only one siRNA resulted in efficient klotho-silencing in HT22, thus, we decided to continue experiments with this siRNA (Figure 1). As assessed by Western Blot method, transmembrane klotho protein level (130 kDa) dropped by 62.13% (*p* < 0.01) in HT-22 hippocampal cells after transfection with klotho siRNA (KLTH-siRNA), when compared to cells treated with negative control siRNA (Ctrl-siRNA). Simultaneously, the pool of the secreted form of klotho protein (65 kDa) was reduced by 80.02% (*p* < 0.01) (Figure 1). 

**Figure 2:**
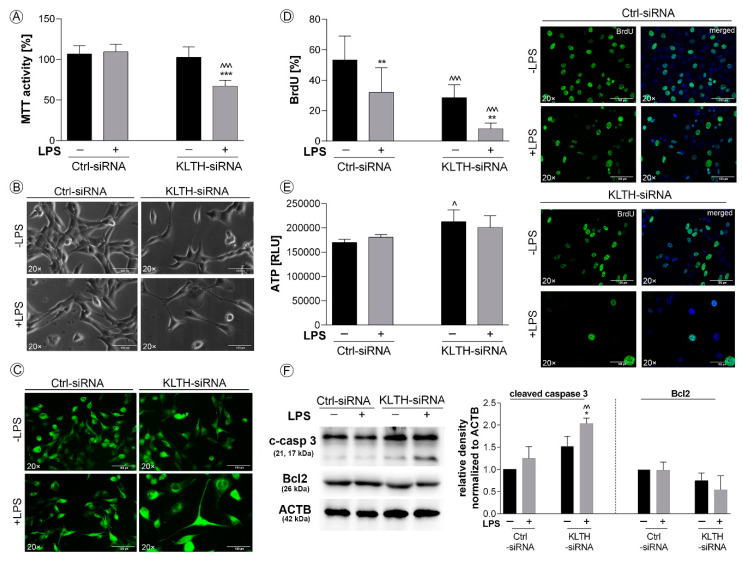
Klotho-depleted HT-22 hippocampal neuronal cells are sensitive to LPS stimulation. The cells were transfected, treated for 48 h with LPS and then, the following parameters were controlled: (**A**) MTT activity; (**B**) morphology; (**C**) cytoskeleton structure; (**D**) BrdU incorporation; (**E**) ATP level; (**F**) expression of proteins associated with apoptosis. Magnification of the objective lens ×20; scale bar, 100 µm. Bars indicate SD, *n* = 3, ***/^^^ *p* < 0.001, **/^^ *p*< 0.01, */^ *p* < 0.05, no indication/no statistical significance (one-way ANOVA and Dunnett’s a posteriori test). (*) indicate comparison between LPS-non-treated and treated Ctrl-siRNA or KLTH-siRNA cells, (^) indicate comparison between LPS-non-treated Ctrl-siRNA and KLTH-siRNA cells or LPS-treated Ctrl-siRNA and KLTH-siRNA cells.

Having established a model of klotho-silencing, we decided to verify whether LPS treatment will affect the general status of HT22 cells. Firstly, klotho-silenced cells were found to be more sensitive to LPS treatment in terms of cell metabolic activity. Detailed analysis revealed a 33.51% reduction in MTT activity in LPS-treated KLTH-siRNA cells when compared to LPS-stimulated control cells (*p* < 0.001) (Figure 2A). As fluctuations in MTT status may result from a reduction in cell number or affected mitochondria condition, in the next part of the study, we controlled both parameters. As shown in Figure 2B, this outcome could be at least partially due to the reduced number of cells. Furthermore, analysis of cellular morphology revealed that KLTH-siRNA cells became flattened, disorganized and enlarged after LPS treatment (Figure 2B). Tubulin staining not only confirmed the reduction in the number of cells and substantial changes in cellular morphology but also fluctuations in cytoskeleton structure (Figure 2C). The reduction of cell numbers resulted from decreased proliferative potential. LPS treatment affected the proliferation potential in control cells and the noted reduction was 39.74% (*p* < 0.01). Further to this, klotho-silencing also resulted in a downregulation of BrdU incorporation by 46.58% (*p* < 0.001) and the observed effect was even more accented after LPS stimulation (*p* < 0.01) (Figure 2D). At the same time, the ATP level reflecting the condition of mitochondria was not associated with LPS. However, a statistically significant slight increase in the ATP pool was observed in HT-22 cells after klotho-silencing (*p* < 0.05) (Figure 2E). Finally, we decided to check whether cells undergo apoptosis and reported an increased level of cleaved (active) caspase 3 in KLTH-siRNA cells challenged with LPS (*p* < 0.01), which was accompanied with a slight drop in Bcl2 pool (*p* > 0.05) (Figure 2F).

### 3.2. Klotho-Depletion Affects Intracellular Redox and Mineral Homeostasis 

**Figure 3:**
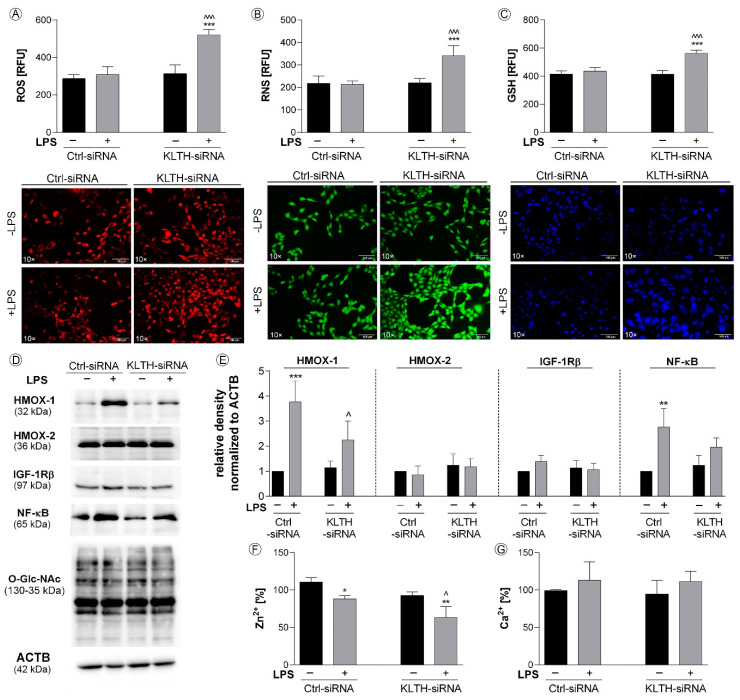
Klotho-depletion affects intracellular redox and mineral homeostasis. The cells were transfected, treated for 48 h with LPS and then, the following parameters were controlled: (**A**) ROS level; (**B**) RNS level; (**C**) GSH level; (**D**–**E**) expression of proteins associated with antioxidant pathways; (**F**) zinc ions level; (**G**) calcium ions level. Magnification of the objective lens ×10; scale bar, 100 µm. Bars indicate SD, *n* = 3, ***/^^^ *p* < 0.001, ** *p* < 0.01, */^ *p* < 0.05, no indication/no statistical significance (one-way ANOVA and Dunnett’s a posteriori test). (*) indicate comparison between LPS-non-treated and treated Ctrl-siRNA or KLTH-siRNA cells, (^) indicate comparison between LPS-non-treated Ctrl-siRNA and KLTH-siRNA cells or LPS-treated Ctrl-siRNA and KLTH-siRNA cells.

LPS treatment promoted the production of total superoxide and nitric oxide in klotho-silenced hippocampal cells, with no effect on control cells. Respectively, a 1.69- (Figure 3A) and 1.60-fold increase (Figure 3B) was reported in LPS-treated KLTH-siRNA cells when compared to LPS-treated Ctrl-siRNA cells (*p* < 0.001). This observation is extremely interesting given the activation of the adaptive response involving the glutathione antioxidant system in KLTH-siRNA cells after LPS stimulation (Figure 3C). On the other hand, LPS treatment led to the activation of NF-κB transcription factor in Ctrl-siRNA but not in KLTH-siRNA cells. Densitometry analysis revealed that the level of NF-κB was augmented by 2.76 times (*p* < 0.01) (Figure 3D,E). Additionally, LPS promoted statistically significant upregulation of heme oxygenase 1 (HMOX-1) in control cells (*p* < 0.001) (Figure 3D,E). However, the expression of heme oxygenase 2 (HMOX-2), considered as constitutively expressed, remained unchanged (*p* > 0.05) (Figure 3D,E). The activation of antioxidants prevented changes in the O-GlcNAcylation pattern in all experimental set-ups analyzed (Figure 3D,E). As oxidative and nitrosative imbalance may affect mineral homeostasis, in the next part of this work, we controlled the intracellular levels of zinc (Figure 3F) and calcium ions (Figure 3G). While Ca^2+^ pools remained unaffected in Ctrl-siRNA and KLTH-siRNA cells even after LPS treatment (Figure 3G), Zn^2+^ levels were downregulated in Ctrl-siRNA and KLTH-siRNA cells as a consequence of LPS stimulation (*p* < 0.05) (Figure 3F). Further to this, we confirmed a reduction in zinc ion levels in LPS-treated KLTH-cells, when compared to Ctrl-siRNA cells, with the noted reduction of 28.34% (*p* < 0.05) (Figure 3F).

### 3.3. Klotho-Silencing Results in Oxidative-Mediated DNA and Telomere Instability

**Figure 4:**
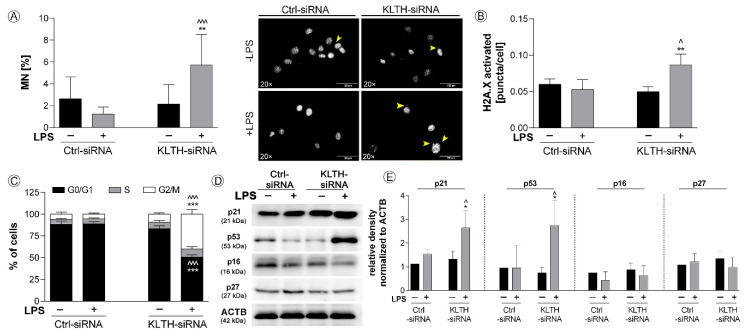

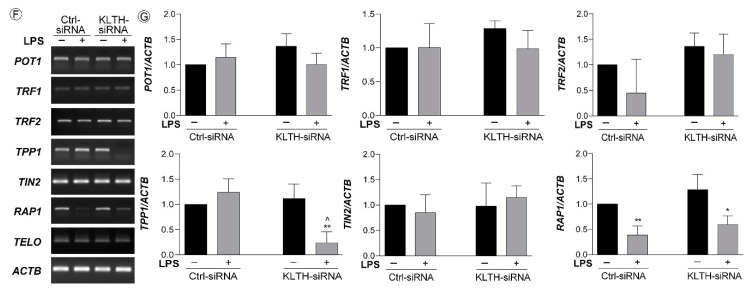
Klotho-silencing results in oxidative-mediated DNA and telomere instability. The cells were transfected, treated for 48 h with LPS and then, the following parameters were controlled: (**A**) micronuclei formation; (**B**) H2A.X phosphorylation; (**C**) cell cycle profile; (**D**–**E**) expression of proteins associated with cell cycle arrest; (**F**–**G**) expression of genes of shelterin complex. Yellow arrows indicate micronuclei. Magnification of the objective lens × 20; scale bar, 100 µm. Bars indicate SD, *n*= 3, ^^^ *p* < 0.001, ** *p* < 0.01, */^ *p* < 0.05, no indication/no statistical significance (one-way ANOVA and Dunnett’s a posteriori test). (*) indicate comparison between LPS-non-treated and treated Ctrl-siRNA or KLTH-siRNA cells, (^) indicate comparison between LPS-non-treated Ctrl-siRNA and KLTH-siRNA cells or LPS-treated Ctrl-siRNA and KLTH-siRNA cells.

We found that klotho-depleted HT-22 cells are more prone to DNA damage and micronuclei formation than control cells. After LPS treatment, micronuclei production was elevated 4.65-fold in KLTH-siRNA cells compared to Ctrl-siRNA cells (*p* < 0.001) (Figure 4A). DNA damage response was also analyzed and a 1.64-fold increase in H2AX phosphorylation status was reported for LPS-stimulated KLTH-siRNA (*p* < 0.05) (Figure 4B). In response to DNA damage, cell cycle arrest was initiated in KLTH-siRNA cells after LPS treatment. The percentage of cells in the G2/M in LPS-treated control cells was 5.36% and the effect was deepened to 40.06% due to klotho-silencing (*p* < 0.001). The effect was accompanied by a drop in the population of cells in G0/G1 cell cycle phase (Figure 4C). The cell cycle arrest was mediated by elevated levels of cell cycle inhibitors, namely p53 and p21. Western Blot analyses revealed that in KLTH-depleted cells, LPS treatment led to 2.91- and 1.72-fold augmentation in the levels of p53 and p21 proteins, respectively, when compared to LPS-treated control cells (*p* < 0.01) (Figure 4D,E). The same analysis confirmed that the levels of p27, as well as p16, remained unaffected in all experimental set-ups analyzed (Figure 4D,E). As cell cycle arrest may result from general DNA damage but also telomere-associated damage, in the next step, we decided to control if klotho-silencing may lead to any changes in telomeric complex and telomere length during incubation with LPS. Firstly, we verified the expression profile of six genes, i.e., POT1, TRF1, TRF2, TPP1, TIN2, and RAP1, involved in the formation of the shelterin complex (Figure 4F,G). Except for RAP1, their expression pattern in Ctrl-siRNA cells was not affected after LPS treatment. Similarly, no fluctuations in the expression of TIN2, POT1, TRF1 and TRF2 in KLTH-siRNA cells due to LPS stimulation were detected. However, a comparison of LPS-treated control and klotho-depleted cells revealed a statistically significant decrease in TPP1 expression (*p* < 0.05). Additionally, the downregulation of RAP1 was comparable in KLTH-depleted HT-22 cells as well as control cells after incubation with LPS (*p* < 0.05) (Figure 4F,G). Finally, the changes in the expression of genes associated with the shelterin complex did not affect the length of telomeres (Figure 4F,G). 

### 3.4. Klotho-Depletion Affects the Inflammatory Response in HT-22 Hippocampal Neuronal Cells 

**Figure 5:**
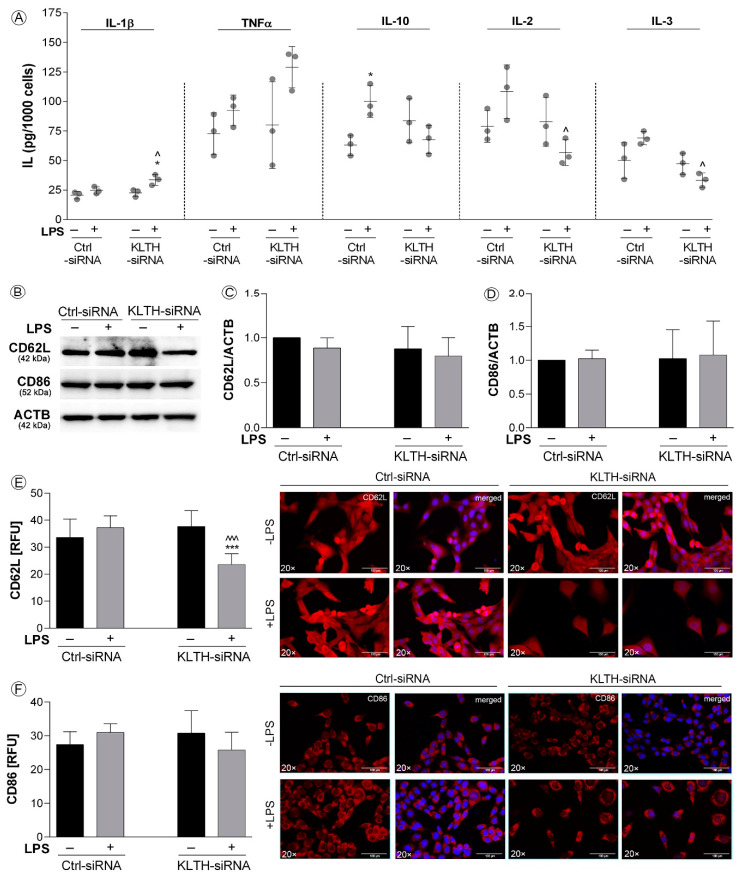

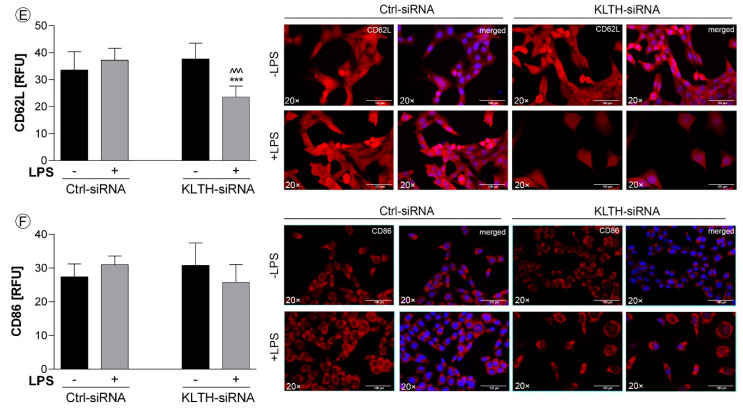
Klotho-depletion affects the inflammatory response in HT-22 hippocampal neuronal cells. The cells were transfected, treated for 48 h with LPS and then, the following parameters were controlled: (**A**) interleukins secretion; (**B**–**D**) expression of proteins CD62L and CD86; (**E**) surface expression of CD62L; (**F**) surface expression of CD86. Magnification of the objective lens ×20; scale bar, 100 µm. Bars indicate SD, *n* = 3, ***/^^^ *p* < 0.001, */^ *p* < 0.05, no indication/no statistical significance (one-way ANOVA and Dunnett’s a posteriori test). (*) indicate comparison between LPS-non-treated and treated Ctrl-siRNA or KLTH-siRNA cells, (^) indicate comparison between LPS-non treated Ctrl-siRNA and KLTH-siRNA cells or LPS-treated Ctrl-siRNA and KLTH-siRNA cells.

Klotho-silencing sensitized HT-22 cells to LPS treatment in terms of inflammatory response. ELISA tests revealed increased secretion of IL-1β in KLTH-cells after LPS treatment (*p* < 0.05) (Figure 5A). Similarly, the level of TNFα was elevated, however, the result did not turn out statistically significant (*p* > 0.05) (Figure 5A). On the other hand, levels of IL-10 were increased in Ctrl-cells (*p* < 0.05), but not in KLTH-cells in response to LPS treatment (Figure 5A). Ctrl-cells did not respond to LPS stimulation in terms of IL-2 and IL-3 release, but significantly lower levels were noted for KLTH-cells (*p* < 0.01) (Figure 5A). Then, using Western Blot method, we controlled the total levels of two crucial proteins involved in an adaptive response to bacterial antigens such as LPS, namely CD62L and CD86. The analysis confirmed a slight decrease in CD62L pool in KLTH-siRNA cells after LPS treatment; however, the result did not turn statistically significant (Figure 5B,C). Furthermore, either LPS treatment or klotho-silencing did not affect the levels of CD86 (Figure 5B,D). At the same time, we decided to control the surface expression of CD62L and CD86 molecules. Extracellular immunostaining revealed that in KLTH-cells, in contrast to Ctrl-siRNA cells, the level of extracellularly expressed CD62L decreased after LPS treatment (*p* < 0.001) (Figure 5E). The same tendency was not observed in the case of CD86 molecule (Figure 5F). 

### 3.5. Klotho-Silencing Promotes Activation of ER Stress Response But Not Autophagy

**Figure 6:**
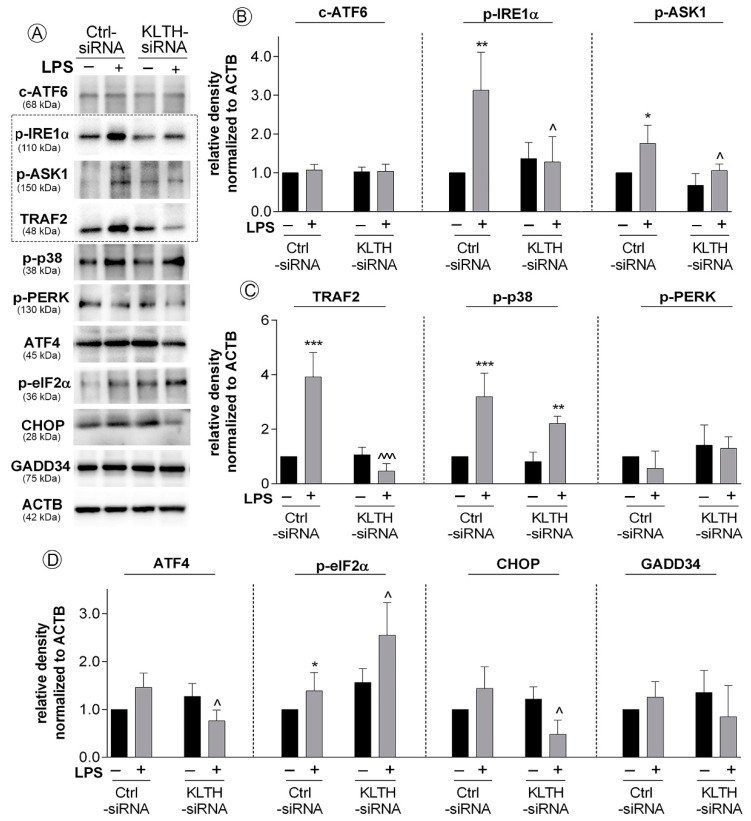
Klotho-silencing promotes activation of ER stress response but not autophagy. The cells were transfected, treated for 48 h with LPS and then, the following parameters: (**A**–**D**) expression of proteins involved in ER stress response was controlled. Bars indicate SD, *n* = 3, ***/^^^ *p* < 0.001, ** *p* < 0.01, */^ *p* < 0.05, no indication/no statistical significance (one-way ANOVA and Dunnett’s a posteriori test). (*) indicate comparison between LPS-non-treated and treated Ctrl-siRNA or KLTH-siRNA cells, (^) indicate comparison between LPS-non treated Ctrl-siRNA and KLTH-siRNA cells or LPS-treated Ctrl-siRNA and KLTH-siRNA cells.

Then, we analyzed the course of three pathways of the endoplasmic reticulum stress response and found activation on one of them. In detail, the fraction of cleaved ATF6 was not affected in any of the experimental set-ups tested (Figure 6A,B). Further to this, we controlled the initiation of the next pathway of ER stress response controlled by IRE1α sensor molecule and followed by the formation of pIRE1α-pASK1-TRAF2 complex. Here, we confirmed LPS-induced enhanced phosphorylation of IRE1α in Ctrl-siRNA cells (3.19-fold increase, *p* < 0.01) (Figure 6A,B). A similar observation was done for phosphorylated ASK1 with 1.76-fold increase when compared to non-treated Ctrl-siRNA cells (*p* < 0.05) (Figure 6A,B). Finally, the pools of TRAF2 were also significantly augmented in Ctrl-siRNA cells due to the LPS stimulation (3.92-fold increase, *p* < 0.001). At the same time, in KLTH-cells, the levels of analyzed factors remained unchanged even after LPS treatment (Figure 6A–C). Activation of pIRE1α -pASK1-TRAF2 complex was followed by phosphorylation of p38 protein in control cells. Interestingly, incubation of KLTH-siRNA cells with LPS also resulted in upregulated levels of p-p38. The observed differences were 3.20- (*p* < 0.001) and 2.72-fold (*p* < 0.01) increases in control and klotho-deficient cells, respectively (Figure 6A,C). We also detected the decreased expression of CHOP, a target molecule of IRE1α pathway, but only in LPS-treated KLTH-siRNA cells (*p* < 0.05) (Figure 6A,D). Furthermore, the phosphorylation pattern of the upstream transcription factor of the third pathway analyzed, i.e., PERK, was not affected in all experimental set-ups analyzed (Figure 6A,C). However, we confirmed activation of its downstream molecule, eIF2α in LPS-treated control HT-22 cells (1.39-fold increase in the pool of phosphorylated fraction when compared to non-treated cells; *p* < 0.05) and the effect was even more pronounced after klotho-silencing (*p* < 0.05) (Figure 6A,D). 

**Figure 7:**
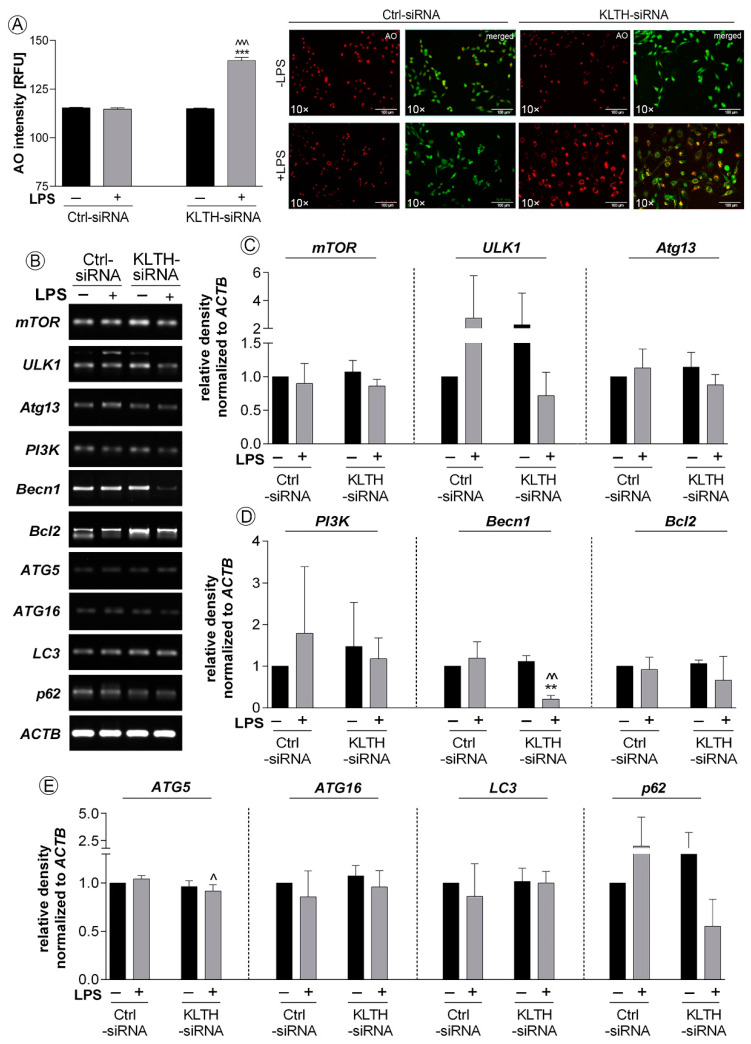
Klotho-silencing promotes activation of ER stress response but not autophagy. The cells were transfected, treated for 48 h with LPS and then: (**A**) presence of acidic compartments and (**B**–**E**) expression of genes involved in autophagy pathway were controlled. Magnification of the objective lens ×10; scale bar, 100 µm. Bars indicate SD, *n* = 3, ***/^^^ *p* < 0.001, **/^^ *p* < 0.01, ^ *p* < 0.05, no indication/no statistical significance (one-way ANOVA and Dunnett’s a posteriori test). (*) indicate comparison between LPS-non-treated and treated Ctrl-siRNA or KLTH-siRNA cells, (^) indicate comparison between LPS-non treated Ctrl-siRNA and KLTH-siRNA cells or LPS-treated Ctrl-siRNA and KLTH-siRNA cells.

Then, we decided to control if perturbations in ER homeostasis result in activation of autophagy in LPS treated control and klotho-deficient hippocampal cells. Firstly, we confirmed a 21.55% increase in acridine orange staining intensity reflecting acidic compartments in LPS-treated KLTH-siRNA cells (*p* < 0.001) (Figure 7A). These experiments were followed by an analysis of expression profiles of 10 genes involved in autophagy initiation and propagation. We found a significant downregulation of expression of two genes i.e., Becn1 and Atg5 in KLTH-siRNA cells after LPS treatment when compared to LPS-treated control cells. The observed reduction was by 82.35% (*p* < 0.01) and 12.50% (*p* < 0.05), respectively (Figure 7B–D). The levels of mTOR, ULK1, Atg13, PI3K, Bcl2, ATG16, LC3 and p62 remained unaffected in all experimental set-ups analyzed (Figure 7B–D).

## 4. Discussion

Previous studies demonstrated that LPS does not impair hippocampal neuronal cells directly but induces neuroinflammation by firstly activating microglia [24]. The fact that hippocampal cells are slightly sensitive to LPS treatment per se results from the low expression of toll-like receptor 4 (TLR4), a molecule crucial for LPS antigen recognition and signal transduction. That is, at least partially, a consequence of the high level of klotho, which was shown to degrade TLR4 via deglycosylation [25]. Klotho-depletion elevates TLR4 levels, and thus, sensitizes HT-22 hippocampal neuronal cells to ER-mediated neuroinflammation as demonstrated in our study. 

**Figure 8:**
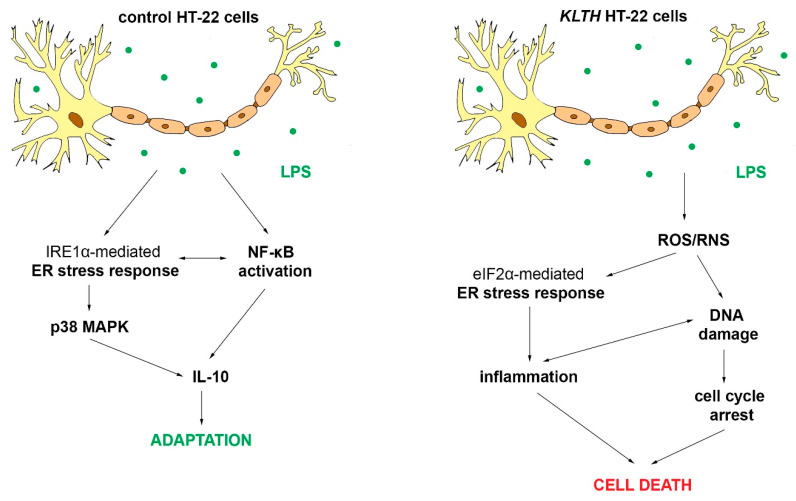
Role of klotho protein in LPS-mediated inflammation in HT-22 hippocampal neuronal cells.

To date, it was shown that the activation of the unfolded protein response (UPR) related to ER stress in neuroglia and neurons is one of the pathological hallmarks of many neurodegenerative diseases. The UPR leads to changes in activities of key modulators, integrating pro-death and pro-survival signals and functions, thus determining cell fate [26]. As shown in this study, klotho functions as a signal transducer between pro-survival and pro-apoptotic crosstalk mediated by ER stress in neurons during the LPS challenge. Firstly, we provide evidence of LPS-mediated activation of antioxidant pathways followed by the initiation of pro-survival endoplasmic reticulum stress response pathways in control hippocampal neurons. In detail, LPS treatment resulted in activation of the major sensor of UPR, i.e., the inositol requiring enzyme 1 α (IRE1 α), and further formation of IRE1 α/TRAF2 complex followed by activation of NF-κB and mitogen-activated protein kinase (MAPK) signaling (Figure 6). This signal was probably initiated by c-Jun N-terminal kinase (JNK) due to its abrogation of a potentially apoptotic signal mediated by the p38 MAPK pathway [27]. In turn, p38 enhanced secretion of a powerful anti-inflammatory cytokine IL-10 (Figure 5) and fulfilled its function in the resolution of inflammation [28]. IL-10 may further act in the positive feedback loop by upregulating extracellular signal-regulated kinase 1/2 phosphorylation (ERK 1/2 MAPK) and downregulation of IKK phosphorylation [29]. Finally, IL-10 exerts beneficial effects in neuronal injury by enhancing expression of Bcl-2 and Bcl-xL, blocking cytochrome c release and caspase cleavage, and activating the canonical NF-κB pathway upregulated translocation of p50, playing a more prominent role in neuronal survival [30]. As an intrinsic consequence of low-level activation of UPR, neurons initiated a predominantly adaptive response, preventing the cell population from succumbing to apoptosis, restoring proliferation, and desensitizing cells to the oxidative stress [31]. Secondly, klotho-depletion prevented the formation of IRE1 α/TRAF2 complex and redirected HT-22 cell fate upon activation of PERK-independent phosphorylation of eIF2α. The PERK-independence is crucial in this action since the PERK-eIF2α arm of URP is considered pro-survival via the translational upregulation of ATF4, resulting in the transcriptional induction of genes encoding antioxidant proteins [32] (Figure 6). However, when acting alone, as observed in this study, eIF2α phosphorylation effectively inhibits cyclin D1 synthesis [33], which was correlated with oxidative stress-mediated p21/p52-dependent G2/M cell cycle arrest in klotho-deficient neurons (Figure 4). The role of the klotho protein as a humoral factor conferring oxidative stress resistance in neuronal cells was well established [34,35,36]. It was shown to enhance the expression of the thioredoxin/ peroxiredoxin system by sustained inhibitory phosphorylation of FoxO3a [37], a transcription factor involved in the negative regulation of NF-κB signaling. This observation is in agreement with our study, where klotho-deficient HT-22 cells did not exhibit a modulated pattern of the NF-κB pathway course even after LPS treatment. Furthermore, the level of oxidative stress was even aggravated by reduced levels of zinc ions, an important co-factor for enzymes of the antioxidant defense system. Downregulated levels of zinc fraction explain also affected glutathione metabolism and partially caspase 3 cleavage in klotho-depleted cells [38] (Figure 3). However, activation of caspase cascade is inseparably linked with irreversible apoptotic cell death. Then, affected redox and mineral homeostasis led in klotho-silenced HT-22 hippocampal neuronal cells to global and telomeric DNA damage. Reported telomere damage was associated with reduced expression of TTP1, a telomere-binding protein responsible for recruiting telomerase to telomeres and their synthesis. As hippocampal neural stem cells highly express telomerase to maintain genomic stability as well as short telomeres being characteristic for neurodegenerative disorders [39], this observation provides a direct link on the line klotho–TPP1–telomere protection. It is worth pointing out that in this study, we did not detect any changes in telomere length, perhaps due to the short experimental time. Continuing, in response to DNA damage, klotho-deficient HT-22 cells activated p21/p53-dependent G2/M cell cycle arrest as well as DNA repair mechanisms (Figure 4). Transcriptional upregulation of p21, in this case, could be a consequence of another, distinct from p53-dependent, mechanism engaging GCN2-eIF2α-p21 pathway [40]. In addition, growth arrest may result from ER stress-induced regulation of G2/M transition through the eIF2α signaling pathway [41]. This finds confirmation in observations on DNA damage-mediated tubular endoplasmic reticulum extension, promoting apoptosis by the facilitation of ER-mitochondria signaling [42]. Furthermore, the elevated phosphorylation status of eIF2α was accompanied by inhibition of CHOP, a factor activating transcription of murine double minute 2 that targets p53 for degradation and is required for neuroprotection [43]. CHOP was also shown to function as a switch between apoptosis and autophagy. In contrast to our study, other data suggest that CHOP knockdown favors ER stress-induced inhibition of apoptosis towards the activation of autophagy. Since the key signal in this crosstalk is a significant expression of ATF4 [44] and in klotho-deficient HT-22 cells, we detected reduced levels of ATF4, we believe that this gives the impulse for selection of apoptosis over autophagy despite CHOP downregulation. This idea is supported by the reported decreased pool of Beclin 1, a protein crucial for autophagosome formation and autophagy propagation (Figure 7) [45]. Likewise, this explains the steady levels of calcium ion pools, since CHOP regulates expression of ERO1α, a factor stimulating IP3R-mediated Ca^2+^ release from the ER [46]. Finally, we provide evidence that apoptotic cell death was mediated and/or accompanied by the induction of a strong inflammatory response in klotho-deficient neurons. This phenomenon could be driven by various mechanisms including ROS/RNS-, DNA damage- or eIF2α-mediated inflammation. The first possibility in the context of neuroinflammation has been exclusively reviewed by others [47,48]. Secondly, DNA damage could also lead to inflammation on the line ROS/RNS-DNA damage-inflammation but also by inducing high mobility group box 1 (HMGB1)-generating inflammatory microenvironment in TLR4-responsive cells [49]. Thirdly, the ER stress pathway is linked to inflammatory responses through the activation of key signaling networks including NF-κB and JAK/STAT, leading to the production of cytokines, chemokines, and reactive species. Here, we provide evidence on eIF2α-mediated enhanced secretion of pro-inflammatory IL-1β, TNFα, accompanied by decreased levels of anti-inflammatory IL-3, IL-2 and IL-10 in klotho-silenced neurons. Furthermore, we confirmed the downregulated exposition of CD62L on HT-22 neuronal cells surface (Figure 5). Adhesion molecule CD62L is not required for the activation of T cells but is crucial in processes engaging myelin damage on the effector cells [50]. CD62L inhibited deposition could be a result of downregulated levels of IL-2 followed by ER stress [51]. IL-2 is considered as the main neuroregulator secreted by neurons being implicated in neurodegeneration by stimulating regulatory T cells. Additionally, its levels are decreased in the hippocampus of patients with Alzheimer’s disease [52]. On the other hand, IL-3 was shown to convey neuroprotection in response to mechanical stretch [53] or amyloid β by mechanisms requiring PI3-kinase and Jak/STAT pathway activation but not MAP kinase [54]. Besides, IL-3 depletion induced a decrease of the anti-apoptotic protein Bcl-2 [54]. In contrast, IL-1β mediates inflammation in neurons by stimulating the release of proinflammatory cytokines and growth factors through p38 MAPK, not NF-κB pathways [55], as observed in this study. Enhanced IL-1β secretion depends on the activation of the NLRP3 inflammasome through a mechanism involving reactive oxygen species formation and ER stress [56]. Furthermore, IL-1β modulates TNFα production by altering TNF receptor shedding and surface abundance. In turn, TNFα could induce neurotoxicity by promoting CYLD-RIP1-RIP3-MLKL-mediated necroptosis of hippocampal neurons [57]. Similarly, TNF-α autocrine signaling during ER stress significantly intensifies the apoptotic signals of the UPR [58]. Thus, observed in this study, inflammation in klotho-deficient HT-22 neuronal cells may be considered as a side effect of apoptosis or a factor leading to its activation. As a consequence, resolution fails and temporally-regulated induction of UPR-dependent inflammatory and apoptotic pathways exacerbates neuroinflammation and compromise cell fidelity (Figure 8).

## 5. Conclusions

In conclusion, in this study, we show for the first time that in HT-22 hippocampal neuronal cells klotho-depletion intensifies LPS-induced oxi-nitrosative stress and genomic instability associated with telomere dysfunctions leading to p53/p21-mediated cell cycle arrest and, in consequence, to ER stress, inflammation as well as apoptotic cell death. On the other hand, in control cells, activation of the IRE1α-p38 MAPK pathway leads to increased secretion of anti-inflammatory IL-10, and thus, provides an adaptation mechanism. Therefore, these results indicate that klotho serves as a part of the cellular defense mechanism engaged in the protection of hippocampal neuronal cells against LPS-mediated neuroinflammation and emerging issues linked with neurodegenerative disorders.

## Supplementary Materials

The following are available online at https://www.mdpi.com/2073-4409/9/5/1231/s1, Table S1: Telomere-related primers sequences used in this study, Table S2: Autophagy-related primers sequences used in this study, List of antibodies.

## References

[1] Snow W.M., Albensi B.C. (2016). Neuronal Gene Targets of NF-κB and Their Dysregulation in Alzheimer’s Disease. Front Mol Neurosci.

[2] Liu Y., Teige I., Birnir B., Issazadeh-Navikas S. (2006). Neuron-mediated generation of regulatory T cells from encephalitogenic T cells suppresses EAE. Nat. Med..

[3] Bondy S.C. (2020). Aspects of the immune system that impact brain function. J. Neuroimmunol..

[4] Disabato D.J., Quan N., Godbout J.P. (2016). Neuroinflammation: The devil is in the details. J. Neurochem..

[5] Skaper S., Facci L., Zusso M., Giusti P. (2018). An Inflammation-Centric View of Neurological Disease: Beyond the Neuron. Front. Cell. Neurosci..

[6] Onasanwo S.A., Velagapudi R., El-Bakoush A., Olajide O.A. (2016). Inhibition of neuroinflammation in BV2 microglia by the biflavonoid kolaviron is dependent on the Nrf2/ARE antioxidant protective mechanism. Mol. Cell Biochem..

[7] Liddelow S.A., Guttenplan K.A., Clarke L.E., Bennett F.C., Bohlen C.J., Schirmer L., Bennett M.L., Münch A.E., Chung W.-S., Peterson T.C. (2017). Neurotoxic reactive astrocytes are induced by activated microglia. Nature.

[8] Almeida P.G., Nani J.V., Oses J.P., Brietzke E., Hayashi M. (2020). Neuroinflammation and glial cell activation in mental disorders. Brain Behav. Immun. -Heal..

[9] Rao J.S., Kellom M., Kim H.-W., Rapoport S.I., A Reese E. (2012). Neuroinflammation and Synaptic Loss. Neurochem. Res..

[10] Xu Y., Sun Z. (2015). Molecular basis of Klotho: From gene to function in aging. Endocr. Rev..

[11] Shiozaki M., Yoshimura K., Shibata M., Koike M., Matsuura N., Uchiyama Y., Gotow T. (2008). Morphological and biochemical signs of age-related neurodegenerative changes in klotho mutant mice. Neuroscience.

[12] Zhu L., Stein L.R., Kim D., Ho K., Yu G.-Q., Zhan L., Larsson T.E., Mucke L. (2018). Klotho controls the brain-immune system interface in the choroid plexus. Proc. Natl. Acad. Sci. USA.

[13] Mytych J., Solek P., Koziorowski M. (2018). Klotho modulates ER-mediated signaling crosstalk between prosurvival autophagy and apoptotic cell death during LPS challenge. Apoptosis.

[14] Mytych J., Solek P., Tabecka-Lonczynska A., Koziorowski M. (2019). Klotho-Mediated Changes in Shelterin Complex Promote Cytotoxic Autophagy and Apoptosis in Amitriptyline-Treated Hippocampal Neuronal Cells. Mol. Neurobiol..

[15] Mytych J., Sołek P., Będzińska A., Rusinek K., Warzybok A., Tabęcka-Łonczyńska A., Koziorowski M. (2019). Klotho-mediated changes in the expression of Atg13 alter formation of ULK1 complex and thus initiation of ER- and Golgi-stress response mediated autophagy. Apoptosis.

[16] Zhou Y., Guo X., Chen W., Liu J. (2019). Angelica polysaccharide mitigates lipopolysaccharide-evoked inflammatory injury by regulating microRNA-10a in neuronal cell line HT22. Artif. Cells Nanomed. Biotechnol..

[17] Khan M.S., Muhammad T., Ikram M., Kim M.O. (2019). Dietary supplementation of the antioxidant curcumin halts systemic LPS-induced neuroinflammation-associated neurodegeneration and memory/synaptic impairment via the JNK/NF-κB/Akt signaling pathway in adult rats. Oxid. Med. Cell Longev..

[18] Kim D.-C., Cho K.-H., Ko W., Yoon C.-S., Sohn J.H., Yim J.H., Kim Y.-C., Oh H. (2016). Anti-Inflammatory and Cytoprotective Effects of TMC-256C1 from Marine-Derived Fungus Aspergillus sp. SF-6354 via up-Regulation of Heme Oxygenase-1 in Murine Hippocampal and Microglial Cell Lines. Int. J. Mol. Sci..

[19] Guan F., Zhou X., Li P., Wang Y., Liu M., Li F., Cui Y., Huang T., Yao M., Zhang Y. (2019). MG53 attenuates lipopolysaccharide-induced neurotoxicity and neuroinflammation via inhibiting TLR4/NF-κB pathway in vitro and in vivo. Prog. Neuropsychopharmacol Biol. Psychiatry.

[20] Solek P., Majchrowicz L., Koziorowski M. (2018). Aloe arborescens juice prevents EMF-induced oxidative stress and thus protects from pathophysiology in the male reproductive system in vitro. Environ. Res..

[21] Mytych J., Wos I., Solek P., Koziorowski M. (2017). Protective role of klotho protein on epithelial cells upon co-culture with activated or senescent monocytes. Exp. Cell Res..

[22] Mytych J., Romerowicz-Misielak M., Koziorowski M. (2018). Klotho protects human monocytes from LPS-induced immune impairment associated with immunosenescent-like phenotype. Mol. Cell. Endocrinol..

[23] Mytych J., Solek P., Będzińska A., Rusinek K., Warzybok A., Tabecka-Lonczynska A., Koziorowski M. (2020). Towards Age-Related Anti-Inflammatory Therapy: Klotho Suppresses Activation of ER and Golgi Stress Response in Senescent Monocytes. Cells.

[24] Calvo-Rodriguez M., De La Fuente C., García-Durillo M., Garcia-Rodriguez C., Villalobos C., Núñez L. (2017). Aging and amyloid β oligomers enhance TLR4 expression, LPS-induced Ca2+ responses, and neuron cell death in cultured rat hippocampal neurons. J. Neuroinflammation.

[25] Bi F., Chen F., Li Y., Wei A., Cao W. (2018). Klotho preservation by Rhein promotes toll-like receptor 4 proteolysis and attenuates lipopolysaccharide-induced acute kidney injury. J. Mol. Med..

[26] Sprenkle N.T., Sims S.G., Sanchez C.L., Meares G.P. (2017). Endoplasmic reticulum stress and inflammation in the central nervous system. Mol. Neurodegener..

[27] Svensson C., Part K., Künnis-Beres K., Kaldmäe M., Fernaeus S.Z., Land T. (2011). Pro-survival effects of JNK and p38 MAPK pathways in LPS-induced activation of BV-2 cells. Biochem. Biophys. Res. Commun..

[28] Ananieva O., Darragh J., Johansen C., Carr J.M., McIlrath J., Park J.M., Wingate A., E Monk C., Toth R., Santos S.G. (2008). The kinases MSK1 and MSK2 act as negative regulators of Toll-like receptor signaling. Nat. Immunol..

[29] Dhingra S., Sharma A.K., Arora R.C., Slezak J., Singal P.K. (2009). IL-10 attenuates TNF-alpha-induced NF kappaB pathway activation and cardiomyocyte apoptosis. Cardiovasc. Res..

[30] Zhou Z., Peng X., Insolera R., Fink D.J., Mata M. (2009). Interleukin-10 provides direct trophic support to neurons. J. Neurochem..

[31] Rutkowski D.T., Arnold S.M., Miller C.N., Wu J., Li J., Gunnison K.M., Mori K., Akha A.A.S., Raden D., Kaufman R.J. (2006). Adaptation to ER Stress Is Mediated by Differential Stabilities of Pro-Survival and Pro-Apoptotic mRNAs and Proteins. PLoS Boil..

[32] Krishnamoorthy J., Tenkerian C., Gupta J., Ghaddar N., Wang S., Darini C., Staschke K.A., Ghosh A., Gandin V., Topisirovic I. (2018). Downregulation of PERK activity and eIF2α serine 51 phosphorylation by mTOR complex 1 elicits pro-oxidant and pro-death effects in tuberous sclerosis-deficient cells. Cell Death Dis..

[33] Hamanaka R.B., Bennett B.S., Cullinan S.B., Diehl J.A. (2005). PERK and GCN2 Contribute to eIF2α Phosphorylation and Cell Cycle Arrest after Activation of the Unfolded Protein Response Pathway. Mol. Boil. Cell.

[34] Yamamoto M., Clark J.D., Pastor J.V., Gurnani P., Nandi A., Kurosu H., Miyoshi M., Ogawa Y., Castrillon D.H., Rosenblatt K.P. (2005). Regulation of oxidative stress by the anti-aging hormone klotho. J. Boil. Chem..

[35] Yao Y., Wang Y., Zhang Y., Liu C. (2017). Klotho ameliorates oxidized low density lipoprotein (ox-LDL)-induced oxidative stress via regulating LOX-1 and PI3K/Akt/eNOS pathways. Lipids Heal. Dis..

[36] Xie B., Nie S., Hu G., Xiong L., Hu F., Li M., Peng T., Nie J., He Y. (2019). The involvement of NF-kappaB/Klotho signaling in colorectal cancer cell survival and invasion. Pathol. Oncol. Res..

[37] Zeldich E., Chen C.-D., Colvin T.A., Bove-Fenderson E.A., Liang J., Zhou T.B.T., Harris D., Abraham C.R. (2014). The Neuroprotective Effect of Klotho is Mediated via Regulation of Members of the Redox System. J. Boil. Chem..

[38] Omata Y., Salvador G.A., Supasai S., Keenan A.H., Oteiza P.I. (2013). Decreased Zinc Availability Affects Glutathione Metabolism in Neuronal Cells and in the Developing Brain. Toxicol. Sci..

[39] Franco S., Blasco M.A., Siedlak S.L., Harris P.L., Moreira P.I., Perry G., Smith M.A. (2006). Telomeres and telomerase in Alzheimer’s disease: Epiphenomena or a new focus for therapeutic strategy?. Alzheimers Dement.

[40] Lehman S.L., Cerniglia G.J., Johannes G.J., Ye J., Ryeom S., Koumenis C. (2015). Translational Upregulation of an Individual p21Cip1 Transcript Variant by GCN2 Regulates Cell Proliferation and Survival under Nutrient Stress. PLoS Genet..

[41] Lee D., Hokinson D., Park S., Elvira R., Kusuma F., Lee J.M., Yun M., Lee S.G., Han J. (2019). ER stress induces cell cycle arrest at the G2/M phase through eIF2alpha phosphorylation and GADD45alpha. Int. J. Mol. Sci..

[42] Zheng P., Chen Q., Tian X., Qian N., Chai P., Liu B., Hu J., Blackstone C., Zhu D., Teng J. (2018). DNA damage triggers tubular endoplasmic reticulum extension to promote apoptosis by facilitating ER-mitochondria signaling. Cell Res..

[43] Engel T., Sanz-Rodgriguez A., Jimenez-Mateos E.M., Concannon C.G., Jimenez-Pacheco A., Moran C., Mesuret G., Petit E., Delanty N., Farrell M.A. (2013). CHOP regulates the p53–MDM2 axis and is required for neuronal survival after seizures. Brain.

[44] Matsumoto H., Miyazaki S., Matsuyama S., Takeda M., Kawano M., Nakagawa H., Nishimura K., Matsuo S. (2013). Selection of autophagy or apoptosis in cells exposed to ER-stress depends on ATF4 expression pattern with or without CHOP expression. Boil. Open.

[45] Jia G., Kong R., Ma Z.-B., Han B., Wang Y.-W., Pan S.-H., Li Y.-H., Sun B. (2014). The activation of c-Jun NH2-terminal kinase is required for dihydroartemisinin-induced autophagy in pancreatic cancer cells. J. Exp. Clin. Cancer Res..

[46] Hetz C. (2012). The unfolded protein response: Controlling cell fate decisions under ER stress and beyond. Nat. Rev. Mol. Cell Boil..

[47] Solleiro-Villavicencio H., Rivas-Arancibia S. (2018). Effect of Chronic Oxidative Stress on Neuroinflammatory Response Mediated by CD4+T Cells in Neurodegenerative Diseases. Front. Cell. Neurosci..

[48] Aguilera G., Colín-González A.L., Rangel-López E., Chavarría A., Santamaría A., Rangel E. (2018). Redox Signaling, Neuroinflammation, and Neurodegeneration. Antioxid. Redox Signal..

[49] Kawanishi S., Ohnishi S., Ma N., Hiraku Y., Murata M. (2017). Crosstalk between DNA Damage and Inflammation in the Multiple Steps of Carcinogenesis. Int. J. Mol. Sci..

[50] Grewal I.S., Foellmer H.G., Grewal K.D., Wang H., Lee W.P., Tumas D., Janeway C.A., A Flavell R. (2001). CD62L Is Required on Effector Cells for Local Interactions in the CNS to Cause Myelin Damage in Experimental Allergic Encephalomyelitis. Immunity.

[51] Kamimura D., Bevan M.J. (2008). Endoplasmic Reticulum Stress Regulator XBP-1 Contributes to Effector CD8+ T Cell Differentiation during Acute Infection1. J. Immunol..

[52] Alves S., Churlaud G., Audrain M., Michaelsen-Preusse K., Fol R., Souchet B., Braudeau J., Korte M., Klatzmann D., Cartier N. (2017). Faculty Opinions recommendation of Interleukin-2 improves amyloid pathology, synaptic failure and memory in Alzheimer’s disease mice. Fac. Opin. – Post-Publ. Peer Rev. Biomed. Lit..

[53] Lim J.C., Lu W., Beckel J.M., Mitchell C.H. (2016). Neuronal Release of Cytokine IL-3 Triggered by Mechanosensitive Autostimulation of the P2X7 Receptor Is Neuroprotective. Front. Cell. Neurosci..

[54] Zambrano A., Otth C., Mujica L., Concha I.I., Maccioni R.B. (2007). Interleukin-3 prevents neuronal death induced by amyloid peptide. BMC Neurosci..

[55] Huang Y., Smith D.E., Ibáñez-Sandoval O., Sims J.E., Friedman W.J. (2011). Neuron-specific effects of interleukin-1beta are mediated by a novel isoform of the IL-1 receptor accessory protein. J. Neurosci..

[56] Kim S., Joe Y., Jeong S.O., Zheng M., Back S.H., Park S.W., Chung H.T. (2014). Endoplasmic reticulum stress is sufficient for the induction of IL-1beta production via activation of the NF-kappaB and inflammasome pathways. Innate Immun..

[57] Liu S., Wang X., Li Y., Xu L., Yu X., Ge L., Li J., Zhu Y., He S. (2014). Necroptosis Mediates TNF-Induced Toxicity of Hippocampal Neurons. BioMed Res. Int..

[58] Hu P., Han Z., Couvillon A.D., Kaufman R.J., Exton J.H. (2006). Autocrine Tumor Necrosis Factor Alpha Links Endoplasmic Reticulum Stress to the Membrane Death Receptor Pathway through IRE1α-Mediated NF-κB Activation and Down-Regulation of TRAF2 Expression. Mol. Cell. Boil..

