# Supplementary material for: Focus on the Role of Klotho Protein in Neuro-Immune Interactions in HT-22 Cells Upon LPS Stimulation
Source: Cells. 2020 May 16;9(5):1231. doi: 10.3390/cells9051231 (PMC7290853; doi:10.3390/cells9051231)
Supplement: Supplementary file 1 [file cells-09-01231-s001.pdf]

Antibodies and primers

Antibodies: anti-klotho (1:1000, #PA5-21078; RRID: AB_11153007), anti-p-NF-κB p50 (1:1000, #PA5-37658; RRID: AB_2554266), anti-β-actin (1:10 000, #PA1-16889; RRID: AB_568434), anti-BrdU (1:200, #MA3-071; RRID: AB_10986341), anti-O-GlcNAc (1:5000, #MA1-072; RRID: AB_326364), anti-HMOX-1 (1:1000, #MA1-112; RRID: AB_2536823), anti-HMOX-2 (1:1000, #PA5-19156; RRID: AB_10985656), anti-CD62 (WB: 1:1000, IF: 1:100, #MA1-10259; RRID: AB_11155439), anti-CD86 (WB: 1:1000, IF: 1:100, #MA1-10293; RRID: AB_11153536), anti-p-p38 (1:1000, #PA5-37536, RRID: AB_2554145), anti-TRAF2 (1:1000, #PA5-20193, RRID: AB_11152352), anti-GADD34 (1:1000, #PA1139, RRID: AB_2539894), anti-p-IRE1 α (1:1000, #PA1-16927, RRID: AB_2262241), anti-p-PERK (1:1000, #PA5-40294, RRID: AB_2576881), anti-ATF6 (1:1000, #PA5-68556, RRID: AB_2688633), anti-ATF4 (1:1000, #PA5-36624, RRID: AB_2553621), anti-p-ASK1 (1:1000, #PA5-36619, RRID: AB_2553618), anti-CHOP (1:1000, #PA5-36796, RRID: AB_2553739), anti-p-eIF2a (1:1000, #MA5-15133; RRID: AB_10983400), anti-p16 (1:1000, #PA1-16639; RRID: AB_568662), anti-p21 (1:1000, #701151; RRID:AB_2532411), anti-p27 (1:1000, #PA5-13254; RRID:AB_2078006), anti-p53 (1:1000, #700439; RRID:AB_2532324) (Thermo Fisher Scientific), anti-γH2AX (1:500, #CS208203) (Merck Millipore), anti-active caspase 3 (1:2000, #NBP1-45435, RRID: AB_10008902) (Novus Biologicals), anti-Bcl2 (1:500, #sc-7382; RRID: AB_626736), anti-IGF-IRβ (1:500, #sc-9038; RRID: AB_671793) (Santa Cruz). Secondary antibodies: HRP-conjugated were: anti-mouse (1:40 000, #A9044; RRID: AB_258431), anti-rabbit (1:40 000, #A0545; RRID: AB_10689821) (Sigma), anti-goat (1:5000, #sc-2768; RRID: AB_656964) (Santa Cruz) and fluorochrome-labelled were anti-rabbit Texas Red (1:1000, #T-2767; RRID: AB_2556776) and anti-mouse Cy3 (1:500, #A10521; RRID: AB_2534030) (Thermo Scientific).

Table 1. Telomere-related primers sequences used in this study.

Gene	Primer sequence	Annealing temp. (℃)
TRF1	fwd: TTCCCGAAAGTGGTGGAGTTT<br>rev: TGGCCTTTAGGCTCATACACA	62
TRF2	fwd: CCTCCCAGAAACTCAAGCGG<br>rev: TCTCGTCAACCACAATCTCCT	63
RAP1	fwd: TTCTTTACCAAATTGTGGTGGCT<br>rev: CTCCGACTTGTAGGCTGTGG	62
TPP1	fwd: GGCAAATGCACTTACAACCCT<br>rev: CTCCGAGAGTCTTTCCAGGT	61
TIN2	fwd: AAGAGCATGACCGTCCTCCT<br>rev: GGGTGGTGTACTTAGTGTCCTC	63
POT1	fwd: TTGGTTTCAACAGCTCCCTATAC<br>rev: GGAGGGCTTCATAGTTTCCACT	62
TELO	fwd: CGGTTTGTTTGGGTTTGGGTTTGGGTTTGGGTTTGGGTT<br>rev: GGCTTGCCTTACCCTTACCCTTACCCTTACCCTTACCCT	72
ACTB	fwd: GCAGGAGTACGATGAGTCCG<br>rev: ACGCAGCTCAGTAACAGTCC	68

Table 2. Autophagy-related primers sequences used in this study.

Gene	Primer sequence	Annealing temp. (℃)
mTOR	fwd: ACCGGCACACATTTGAAGAAG<br>rev: CACCACCAAGGATAAGGTAG	52.4
ULK1	fwd: AGGATGGGGACTTGGTTGC<br>rev: CGATGTTTTCGTGCTTTAGTTCC	52.4
PI3K	fwd: CCTGGACATCAACGTGCAG<br>rev: TGTCTCTTGGTATAGCCCAGAAA	53.2
BECN1	fwd: AGTTGAGAAAGGCGAGACAC<br>rev: CACCACCAAGGATAAGGTAG	54.4
BECN2	fwd: GTCGCTACCGTCGTGACTTC<br>rev: CAGACATGCACCTACCCAGC	55.9
ATG16	fwd: CAGAGCAGCTACTAAGCGACT<br>rev: AAAAGGGGAGATTCGGACAGA	52.4
LC3	fwd: CGAGAGCAGCATCCTACCAA<br>rev: TTCTTCCGCGAATGTCGAGT	55.3
ATG13	fwd: CAGAACTGCTGGTGAGGACACT<br>rev: AGCAGGCTGATAGGAAAGGCGA	56.7
ATG5	fwd: AGCAACTCTGGATGGGATTG<br>rev: CACTGCAGAGGTGTTTCCAA	51.8
ACTB	fwd: CATCGGCAATGAGCGGTTCC<br>rev: CCGTGTTGGCGTAGAGGTCC	68.1
